# Resiniferatoxin: The Evolution of the “Molecular Scalpel” for Chronic Pain Relief

**DOI:** 10.3390/ph9030047

**Published:** 2016-08-11

**Authors:** Dorothy Cimino Brown

**Affiliations:** Department of Clinical Studies—Philadelphia, School of Veterinary Medicine, University of Pennsylvania, Philadelphia, PA 19104, USA; dottie@vet.upenn.edu; Tel.: +1-215-898-0030

**Keywords:** TRPV1, resiniferatoxin, chronic pain, analgesia

## Abstract

Control of chronic pain is frequently inadequate or can be associated with debilitating side effects. Ablation of certain nociceptive neurons, while retaining all other sensory modalities and motor function, represents a new therapeutic approach to controlling severe pain while avoiding off-target side effects. transient receptor potential cation channel subfamily V member 1 (TRPV1) is a calcium permeable nonselective cation channel expressed on the peripheral and central terminals of small-diameter sensory neurons. Highly selective chemoablation of TRPV1-containing peripheral nerve endings, or the entire TRPV1-expressing neuron itself, can be used to control chronic pain. Administration of the potent TRPV1 agonist resiniferatoxin (RTX) to neuronal perikarya or nerve terminals induces calcium cytotoxicity and selective lesioning of the TRPV1-expressing nociceptive primary afferent population. This selective neuroablation has been coined “molecular neurosurgery” and has the advantage of sparing motor, proprioceptive, and other somatosensory functions that are so important for coordinated movement, performing activities of daily living, and maintaining quality of life. This review examines the mechanisms and preclinical data underlying the therapeutic use of RTX and examples of such use for the management of chronic pain in clinical veterinary and human pain states.

## 1. Introduction

Nearly two decades of preclinical research supports the involvement of thermoTRP channels in nociceptive transmission and sensitization. The identification of potent, subtype selective agonists and antagonists that demonstrate attractive preclinical pharmacological activity have generated extensive pharmaceutical interest for effective management of chronic pain patients without the need for long-term use of opioid or non-steroidal anti-inflammatory drugs. transient receptor potential cation channel subfamily V member 1 (TRPV1) is the most extensively studied mammalian transient receptor potential channel (TRP) channel with the potential for life changing pain control, particularly in patients with intractable chronic pain states [1,2,3,4,5].

TRPV1 is a calcium permeable non-selective cation channel with expression restricted to small and medium sized sensory neurons in the dorsal root, trigeminal, and vagal ganglia [6,7,8]. These neurons form the unmyelinated or thinly myelinated C and Aδ sensory nerve fibers that project to most organs and tissues. Functionally, TRPV1 acts as a sensor for noxious heat (>~42 °C) and can also be activated by acidic solutions (pH < 6.5), as well as some endogenous lipid-derived molecules. However, it is the discovery of the agonist actions of some plant derived compounds such as capsaicin and resiniferatoxin (RTX) that are the focus of pharmaceutical development for long-term chronic pain relief. The understanding of the mode of action of the known TRPV1 ligands including RTX is a continually evolving area of research and is pivotal to a successful drug design approach to the management of chronic pain [9,10,11,12].

Resiniferatoxin, derived from the *Euphorbia resinifera* plant, is the most potent amongst all known endogenous and synthetic agonists for TRPV1. RTX causes extremely prolonged channel opening and calcium influx, which results in cytotoxicity to the TRPV1-positive pain fibers or cell bodies [13]. When applied to the sensory neuron perikarya, the prolonged calcium influx induced by RTX specifically deletes only the sensory neurons that express the TRPV1 ion channel. Thus, intrathecal and intraganglionic RTX administration leads to selective targeting and permanent deletion of the TRPV1-expressing Aδ and C-fiber neuronal cell bodies in the dorsal root ganglia (DRG) [14,15]. Loss of these sensory neurons interrupts the transmission of pain information from the body to second-order spinal cord neurons, which in turn convey the information to the brain. At the same time, noxious and nonnoxious mechanosensation, proprioception, and locomotor capability are retained [14]. This highly selective chemoablation of specific neurons has been coined “molecular neurosurgery” and RTX coined a “molecular scalpel” in this novel approach that is under study for the permanent control of chronic pain [16].

This review examines the development of RTX to control intractable pain conditions through the permanent deletion of TRPV1 positive sensory nerve fibers. It describes the main studies on RTX mechanism of action and the animal translational research that formed the basis of the human clinical trial currently underway evaluating the use of intrathecal RTX for intractable pain in patients with advanced cancer.

## 2. Resiniferatoxin Is a Mechanism Based Treatment for Chronic Pain

The vision to develop RTX for control of intractable pain chronic pain states began with studies utilizing live cell microscopic evaluation of stably transfected cell lines expressing a TRPV1eGFP fusion protein [17,18]. Olah et al. used ratiometric imaging of intracellular free calcium and confocal imaging of the TRPV1-green fluorescent fusion protein to demonstrate that the endocannabinoid anandamide, could induce an elevation of intracellular free calcium, resulting in intracellular membrane changes in DRG neurons or transfected cells expressing TRPV1. The subsequent fragmentation of the endoplasmic reticulum and mitochondria could result in intracellular dysfunction and axonal damage of TRPV1-positive DRG neurons. If the cell bodies of nociceptors were exposed to anandamide, cell death could ensue through toxic accumulation of calcium [17].

As an ultrapotent vanilloid, RTX, was shown to bind with nanomolar affinity to TRPV1 or TRPV1eGFP positive cells causing prolonged opening of the TRPV1 ion channel with a rapid and massive increase in intracellular calcium [13]. Confocal imaging revealed that within 1 min, RTX induced vesiculation of the mitochondria and the endoplasmic reticulum of the nociceptive primary sensory neurons endogenously expressing TRPV1 due to a sudden increase in calcium. Within 5–10 min nuclear membrane disruption occurred, and cell lysis was documented within 1–2 h followed by specific deletion of TRPV1-expressing cells [18].

Importantly, the presence of TRPV1 is critical for this RTX action. Without expression of TRPV1, RTX at concentrations 1000 times above the dose used to lesion expressing cells, nerve terminals or axons does not appear to produce any negative effects on non-TRPV1-expressing cells at the cellular level. Non-expressing neurons appear to remain intact even when they are adjacent to TRPV1 expressing neurons that are undergoing damage from RTX activation. These conclusions were reached using the live cell imaging of cultured DRG neurons and the imaging of cells transiently and stably transfected with TRPV1 in the studies described above. In addition, Caudle et al demonstrated that RTX application to DRG cells known to express V1, induced large inward currents that were not induced in DRG cells that do not express the receptor [19].

These data demonstrate that vanilloids can disrupt vital organelles within the cell body of sensory ganglia and RTX, as an ultrapotent vanilloid, might be used to rapidly and selectively delete nociceptive neurons. The next step towards the goal of using RTX in the clinical setting to elicit sustained pain relief was to demonstrate the histopathological and behavioral effects of deletion of subpopulations of primary afferent pain-sensing neurons via central administration of RTX into the cerebrospinal fluid or ganglia in vivo.

## 3. Preclinical Studies in Laboratory Animals

Sensory neurons in the dorsal root and trigeminal ganglia collect noxious information from well-defined anatomic areas throughout the body. Targeting specific ganglia for treatment with RTX could, therefore, offer relief of well-localized pain. To ablate the sensory neurons, RTX needs to be injected centrally, either directly into the sensory ganglia or administered intrathecally to target the ganglionic nerve roots. Both approaches have been investigated in animal models.

### 3.1. Corneal Application of Capsaicin

In rats, RTX can be microinjected unilaterally into the trigeminal ganglia using a transcranial stereotaxic approach [14] or a percutaneous approach using an electrical-stimulation needle inserted through the infraorbital foramen [20]. Then, 24 h after injection, an analgesic effect can be documented using the eye-wipe response to corneal application of capsaicin, which is a sensitive test for C-fiber function. Complete suppression of the eye-wiping response evoked by intraocular capsaicin drops was obtained with 200 ng of RTX. The eye-wiping response did not return by the termination of the experiments 350 days after injection suggesting a permanent antinociceptive effect from a single intraganglionic injection. In a similar study, 20 µL RTX solution (0.1 mg/mL concentration) was infused unilaterally in nonhuman primate trigeminal ganglia [21]. Animals were tested for number of eye blinks, eye wipes, and duration of squinting in response to the corneal application of capsaicin for up to 12 weeks after RTX infusion. As it was documented in the rats, the response to capsaicin stimulation in the monkeys was selectively and significantly reduced throughout the duration of the study. The eye-wipe response to corneal application of capsaicin has also been used to document the selective deletion of TRPV1 positive sensory neurons in C57BL/6J mice [22]. The amount of 100 μg RTX or vehicle alone was injected into the cerebrospinal fluid at the cisterna magna. The RTX-treated mice were completely insensitive to the corneal application of capsaicin, while vehicle-treated C57BL/6J mice had a response similar to non-treated animals.

The selectivity of this analgesic approach of sensory neuron ablation was demonstrated with immunohistochemical analysis of the RTX-treated ganglia showing selective elimination of TRPV1-positive neurons. The animals showed no neurological deficits or signs of toxicity. There were no corneal damage or observable alterations in eating or grooming habits that might indicate the presence of a sensory dysesthesia and the loss of capsaicin chemosensitivity did not affect the mechanosensitive response of the corneal reflex. The animals blinked momentarily in response to the liquid droplet itself touching the eye on application.

### 3.2. Intraplantar Capsaicin and Carrageenan

Following the intrathecal administration of RTX in adult rats, nocifensive behavior was tested with the intraplantar injection of capsaicin [23,24]. A dramatic decrease in pain sensitivity was observed as indicated by reduction in both the duration and number of guarding and licking behaviors exhibited by the rats. The selective alleviation of inflammatory thermal hypersensitivity with intrathecal RTX was also tested [23]. Inflammation was induced by 100 μL of 2% carrageenan in the paw and the intrathecal RTX treated animals showed no change in the paw withdrawal latency due to inflammation.

In these studies, the selectivity of this analgesic approach was demonstrated by further testing, showing that intrathecal RTX did not affect paw withdrawal latency to von Frey filaments after inflammation. This observation is consistent with the notion that RTX treatment does not affect mechanical hypersensitivity due to inflammation because mechanical sensitivity is carried by a distinct set of nociceptors that do not express TRPV1. In addition, immunohistochemistry documented a complete loss of TRPV1 labeling in the dorsal horn of the spinal cord that was localized to the lumbar spinal segments closest to the level of the intrathecal injection [23].

### 3.3. Noxious Thermal Stimulation

In dogs, behavioral testing was performed to establish baseline paw withdrawal latency to a radiant thermal stimulus. The unrestrained animal was placed on a glass-top table, and a focused radiant halogen heat source was positioned under a paw. When the dog lifted its limb, the time in seconds was recorded, and the heat source was terminated. A maximum exposure time of 20 s was allowed to prevent injury to the animal. Under general anesthesia, RTX was then administered intrathecally into the cisterna magna at 0.1, 1.2, or 3 μg/kg. Two days after treatment, the 1.2 and 3.0 μg/kg doses caused nearly complete loss of sensitivity to noxious thermal stimulation. Compared with pretreatment values, limb withdrawal latency was substantially increased, to the point of cutoff. The effect was maintained when behavior testing was repeated 5, 7, 10, and 12 days after RTX administration [25].

On repeated neurologic examinations following injection, no negative effects from intrathecal RTX were documented. All dogs maintained normal locomotor and proprioceptive activities. At necropsy, all gross and histopathologic findings associated with the spinal cord and spinal canal were consistent with intrathecal catheter placement. In addition, blood and urine collected before one and two weeks after RTX administration revealed no significant increases or decreases of parameters out of the normal range.

### 3.4. Operant Orofacial Assay

To account for the fact that pain is ultimately experienced as a culmination of complex information from the periphery, Neubert et al. used an operant orofacial assay to evaluate and characterize thermal pain sensitivity in mice [22]. Operant systems utilize a reward-conflict platform, in which animals choose between receiving a reward or escaping an unpleasant stimulus. The animals control the amount of nociceptive stimulation and modify their behavior based on cerebral processing. The mice completed a reward-conflict task whereby they would contact a thermode with their face to access a reward bottle, generating an electrical signal that was acquired for analysis. They documented that TRPV1 knock out mice were insensitive to noxious heat within the activation range of TRPV1 (37–52 °C) and, in addition, mice treated with intracisternal RTX, had significantly higher licks as compared to the vehicle treated animals when tested with the thermode in the noxious heat range.

While operant assays are not new [26,27] the renewed interest in their use stems from the growing frustration over the mounting failures in translating basic scientific data into clinically available analgesics using conventional animal models. The lack of success has been attributed to both unacceptable side effects and lack of efficacy in humans of interventions that appeared to be safe and effective in animal models [28]. The use of operant measures is suggested as one approach to overcome this translational gap based on several examples of discordance between the results obtained using operant versus reflexive measures in the same study, with the operant measure agreeing with the clinical outcome [29].

In addition to designing studies that use operant as opposed to reflexive outcome measures, a variety of other recommendations have been made in the hope of improving the ability of animal studies to predict clinical trial outcomes. These include the utilization of animal models that are more directly applicable to prevalent painful conditions in people, as well as using outcomes that measure spontaneous behaviors and a broader range of “quality of life” [28,30,31,32]. With these recommendations in mind, the development of intrathecal RTX for intractable chronic pain states moved to studies in companion dogs with the spontaneous development of bone cancer pain.

## 4. Preclinical Studies in Companion Dogs

### 4.1. Rationale

Because studies in laboratory animals, mainly rodents, with experimentally induced pain states have only been partially successful in predicting human clinical trial outcomes, supplementing drug development with additional models can provide an informative transitional step for translating novel treatments to human patients. There is growing interest in using the diseases that spontaneously develop in companion (pet) dogs to investigate efficacy of new pharmacological agents and interventional administration approaches. The interest stems from the fact that:

- The spontaneously developed diseases can be pathologically, physiologically and symptomatically analogous to those in people [32,33,34,35,36].
- Medical surveillance of dogs is second only to that of people and illnesses are managed by veterinary specialists using all of the diagnostic approaches of modern medicine [37].
- Dogs share the environment with people and thus the potential environmental risk factors for disease.
- Their large body size simplifies biologic sampling.
- The extended course of disease, compared to rodent models, allows for clinically relevant efficacy data collection, while the shorter overall lifespan of dogs, compared to humans, provides a time course of disease within a time-frame reasonable for efficient data collection.
- Outcome assessment instruments have been specifically developed to capture clinically and translationally relevant pain severity and pain impact data in these models [38].
- Dogs have significant intrabreed homogeneity coupled with marked interbreed heterogeneity, providing unique opportunities to understand the genetic underpinnings of disease [39].
- The spontaneous pain caused by these naturally occurring diseases requires treatment for the animals’ sake, and carefully studying novel therapies in these dogs can provide greater insight into the potential efficacy in humans [25,40,41].

Keeping in mind that a goal in using RTX in the clinical setting is to elicit sustained pain relief through deletion of subpopulations of primary afferent pain-sensing neurons, particularly in intractable conditions, an obvious target population is the 75% to 90% of patients with advanced cancer that experience significant, life-altering, cancer-induced pain [42]. The most severe pain is especially associated with tumors involving bone destruction. In the evaluation of RTX efficacy for chronic pain, the naturally occurring companion dog model of bone cancer pain has been instrumental in documenting analgesic efficacy and potential side effects; as well as informing future clinical trial design, justifying the starting dose, and selection of outcome measures and primary endpoints [14,25,40,43].

### 4.2. Canine Bone Cancer

**Figure 1:**
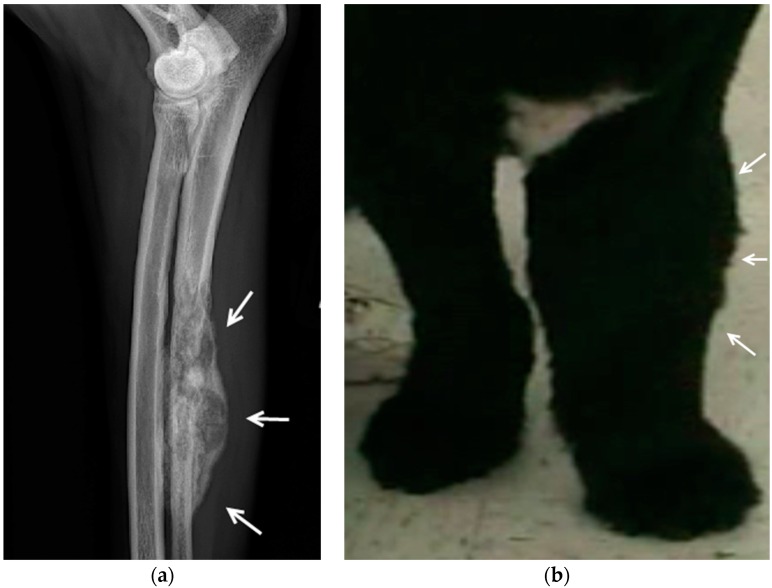
An example of canine bone cancer: (**a**) A radiograph of the left radius and ulna reveals a severe moth eaten osteolytic lesion of the ulna (white arrows); (**b**) Compared to the right forelimb there is marked swelling of the left forelimb (white arrows) due to edema associated with the underlying bone tumor.

The spontaneous development of bone cancer is common in companion dogs and bears striking resemblance to bone cancer in humans (Figure 1). In both species, osteosarcoma is histologically indistinguishable and has the same biologic behavior and disease progression [44]. For a variety of reasons, many owners do not choose the standard-of-care management for their dogs with appendicular osteosarcoma, which is amputation followed by chemotherapy. For these animals, the goal is to maintain the dog’s comfort and quality of life for as long as possible. The issues associated with managing pain in dogs with bone cancer parallel those that occur in human cancer patients. Over time pain severity becomes refractory to conventional pain management as the disease progresses [44,45,46]. Dogs often undergo euthanasia within several months of diagnosis due to uncontrolled bone pain and associated loss of function. This evolution of bone pain over weeks to months allows enough time to evaluate the effectiveness of antinociceptive agents through the evolution of the pain process including episodes of break through pain. This time is still short enough, however, to ensure rapid accrual of data through detailed prospective studies.

#### 4.2.1. Outcomes

In addition to identifying animal models that more closely represent the prevalent human disease conditions, much attention has been paid to the choice of outcomes in animal studies with a call to use outcomes that measure spontaneous behaviors and a broader range of ‘quality of life’ measures as opposed to evoked responses [28,31,32]. In addition to laboratory based measures, such as gait analysis to quantify lameness [47], a variety of clinically relevant outcomes measures have been validated in dogs to capture changes in spontaneous pain-related behaviors and overall quality of life, over extended periods of time, in the animal’s home environment. Watch-sized, accelerometer based activity monitors can be unobtrusively worn on the dogs’ collar to collect activity data for weeks or months at a time, while the dog performs its routine activities of daily living in its home environment. Increased activity levels can be documented in dogs with chronic painful conditions when appropriate anti-inflammatories and analgesics are administered [41,48]. In addition, much like the proxy assessment of pain behaviors in young children or cognitively impaired populations, owner assessments of chronic pain behaviors in their dogs have been validated [49,50,51]. These assessments allow the measurement of pain severity and its impact on the dogs function as well as overall quality of life. In some cases, these measures were developed to specifically to not only reliably quantify chronic pain behaviors in dogs, but also to have translational relevance to human studies [38]. Several of these measures were used to quantify the severity of chronic pain and its impact on the function of dogs treated with intrathecal RTX.

#### 4.2.2. Analgesic Efficacy of RTX

To generate preclinical data supporting the fact that intrathecal RTX could provide effective pain relief and improve function in bone cancer pain, a single-blind, controlled study in 72 companion dogs with bone cancer was implemented [40]. Dogs were randomized to standard of care analgesic therapy alone or 1.2 mg/kg intrathecal RTX in addition to standard of care analgesic therapy. To maintain owner blinding, all dogs were admitted to the hospital for randomization and the fur was clipped over the intravenous catheter and intrathecal injection sites. While only the dogs randomized to the RTX group were anesthetized and underwent intrathecal injection, all dogs were hospitalized overnight to allow treated dogs to fully recover and were discharged the next day from the hospital to owners who were unaware as to which group their dog was randomized. Dogs were evaluated two weeks after the randomization visit and then at monthly intervals until death. Unblinding occurred when an owner believed that their dog had an unacceptable level of discomfort and required an intervention or at the time of spontaneous death or euthanasia of the dog.

Five efficacy outcomes were evaluated in this study. Although both of the primary outcomes and the lameness secondary outcome revealed a positive effect of RTX, the owner pain scores did not. This could be attributable to the loss of study power due to seven dogs in the treated group undergoing euthanasia prior to the two-week endpoint and the incidence of spinal headache in some of the RTX treated dogs. The negative behaviors associated with a spinal headache—lethargy, lack of interaction with the family, and inappetance—in the first week after randomization could influence the owners’ pain assessment. Overall, dogs in the control group required unblinding significantly sooner than dogs that had been treated with RTX. 78% of dogs in the control group required unblinding and adjustment in analgesic protocol or euthanasia within six weeks of randomization, while only 50% of the dogs treated with RTX required unblinding and adjustment in analgesic protocol or euthanasia in that same time frame. Analgesic efficacy was also documented by an orthopedist, blinded to treatment group, who evaluated lameness through video analysis and determined that 7% of dogs in the control group had improved lameness while 33% of dogs in the RTX-treated group had improved lameness two weeks after randomization. While these differences between groups were statistically significant and support the analgesic efficacy of intrathecal RTX in bone cancer pain, it was clear that there was variability in response in the RTX treated dogs.

The variability in the response to RTX may be associated with the varying degree of TRPV1 expression on the small to medium sized DRG neurons that can be documented with immunocytochemical staining [52]. It is possible that high expressing neurons are the most susceptible to RTX cytotoxicity, while lower expressing neurons may be able to sequester a minor influx of calcium and survive transient exposure to RTX, making them less susceptible. Low expressing neurons may remain intact and continue to transmit clinically relevant pain signals and some neurons that are damaged but repair with time may eventually resume transmitting clinically relevant pain signals. In these cases, it is possible that retreatment would lead to a renewed clinical response. Retreatment was offered for dogs that had initially responded to RTX but had recurrence of chronic pain, however the owners opted not to retreat due to the advanced nature of their dogs’ disease.

Upon necropsy, the DRG of treated dogs revealed RTX-related effects. Within one month post injection, degenerating neurons are in the process of being replaced by rosettes of proliferating satellite cells. Neurons with larger cell body diameters remain unaffected, even in the immediate vicinity of a degenerating neuron [25]. These histologic findings reflect the observed, analgesic effect that occurs in the dogs with the retention of other sensory and proprioceptive functions. In fact, the analgesic effect was documented in the dogs with bone cancer without any evidence of development of neurologic abnormalities that can be seen with neurolytic therapies.

#### 4.2.3. Adverse Events

Significant increases in blood pressure and heart rate can occur after intrathecal RTX injection in dogs. These cardiovascular effects peak within minutes of injection and return to baseline over the hour that the dog remains anesthetized through the period of TRPV1 activation. Immediately after extubation, many dogs pant for several hours, during which they develop hypothermia that plateaus 3 to 4 h after extubation. Even the most hypothermic animals—those that drop core body temperature more than 4 °C after injection—otherwise make an uneventful recovery and regain normothermia in 12 to 18 h [40].

A serious adverse event that can be seen with neurolytic therapies is deafferentation pain syndromes. Complete or partial interruption of afferent nerve impulses can lead to central sensitization, with patients experiencing abnormal sensory phenomena such as allodynia, hyperalgesia, dysesthesias, and hyperpathia [53,54]. In animals, deafferentation can lead to self-mutilation, biting the region in which they might feel painful, or paresthetic sensations [55,56,57]. Behaviors consistent with development of deafferentation pain syndromes did not occur in any dogs treated with RTX. The lack of long-term negative effects is important for the clinical translation of RTX. However, there were several acute perinjection effects of RTX noted. Significant increases in blood pressure and heart rate occurred after intrathecal RTX injection in many dogs. These effects typically peak within five min of injection and then gradually return to baseline over the hour that the dog remains anesthetized through the excitation phase of TRPV1 activation. Upon recovery from general anesthesia, many dogs begin panting heavily and can continue to do so for several hours, during which time they tend to become hypothermic. The hypothermia can be significant and persist for many hours, however it does not prohibit the dogs from otherwise making an uneventful recovery [25,40].

While the preclinical laboratory animal studies provided the necessary mechanistic insights into how to use RTX as a therapeutic agent; the canine companion animal bone cancer studies showed that RTX worked well on the complex pain state that develops due to naturally occurring cancer. These studies were the impetus to move intrathecal RTX into human clinical trials.

## 5. The Clinical Trial

Intrathecal RTX is undergoing a Phase I clinical trial to treat refractory severe pain in patients with advanced cancer. Serial electrocardiogram, brain and spine MRI, eye exam, blood analysis and neurological exam and tools measuring pain, quality of life, active and mental status are used to assess patients pre- and post-injection. An intrathecal catheter is placed and the injection performed under anesthesia to prevent the acute pain that accompanies excitotoxic actions of RTX on TRPV1 neurons. To date, patients have received 3 to 26 µg of RTX into the intrathecal space. While patients receiving the lower doses experienced variable amounts of pain relief, those who received either 13 or 26 μg injections of RTX, showed a clinically meaningful improvement in quality of life following the single injection. These patients consistently reported less pain and improved mobility. No changes in EKG, MRI, or eye examination were noted. Thermal perception reduction was consistent with cell death of the TRPV1 neurons. There were no other sensory or motor changes post-treatment. These initial findings suggest that intrathecal RTX administration can selectively delete neurons that transmit pain. Additional accruals will further detail the safety and efficacy of RTX to reduce refractory pain and improve quality of life in patients with advanced cancer [52,58,59].

## 6. Conclusions

The vision to develop RTX for control of intractable pain conditions emerged from live cell imaging documenting that, as an ultra-potent TRPV1 agonist, RTX causes extremely prolonged channel opening and calcium influx resulting in cytotoxicity to the TRPV1-positive pain fibers or cell bodies [13,17,18]. The selectivity and behavioral effects of deletion of subpopulations of primary afferent pain-sensing neurons via central administration of RTX into the cerebrospinal fluid or ganglia was then documented in a variety of animal evoked pain models [14,20,22,23,24,25]. The final impetus to carry intrathecal RTX into human clinical trials emerged from the preclinical companion animal canine bone cancer studies [14,25,40]. These studies showed that this analgesic approach worked, not just in evoked pain models, but also in the complex pain state that originates from naturally occurring cancer. At the conclusion of these studies, the various elements necessary for a Phase I clinical trial in humans were assembled. Several cohorts of patients with advanced cancer have been recruited to date with promising results [58,59]. Additional accruals will further detail the safety and efficacy of RTX to reduce refractory pain and improve quality of life in patients with advanced cancer.
